# Isolated Perfused Rat Livers to Quantify the Pharmacokinetics and Concentrations of Gd-BOPTA

**DOI:** 10.1155/2018/3839108

**Published:** 2018-07-11

**Authors:** Catherine M. Pastor

**Affiliations:** ^1^Department of Radiology, Hôpitaux Universitaires de Genève, Geneva, Switzerland; ^2^Laboratory of Imaging Biomarkers, Centre of Research on Inflammation, UMR 1149 Inserm, University Paris Diderot, Paris, France

## Abstract

With recent advances in liver imaging, the estimation of liver concentrations is now possible following the injection of hepatobiliary contrast agents and radiotracers. However, how these images are generated remains partially unknown. Most experiments that would be helpful to increase this understanding cannot be performed *in vivo*. For these reasons, we investigated the liver distribution of the magnetic resonance (MR) contrast agent gadobenate dimeglumine (Gd-BOPTA, MultiHance®, Bracco Imaging) in isolated perfused rat livers (IPRLs). In IPRL, we developed a new set up that quantifies simultaneously the Gd-BOPTA compartment concentrations and the transfer rates between these compartments. Concentrations were measured either by MR signal intensity or by count rates when the contrast agent was labelled by [^153^Gd]. With this experimental model, we show how the Gd-BOPTA hepatocyte concentrations are modified by temperature and liver flow rates. We define new pharmacokinetic parameters to quantify the canalicular transport of Gd-BOPTA. Finally, we present how transfer rates generate Gd-BOPTA concentrations in rat liver compartments. These findings better explain how liver imaging with hepatobiliary radiotracers and contrast agents is generated and improve the image interpretation by clinicians.

## 1. Introduction

In the past, great achievements were made by analysing the drug plasma concentrations to understand their body distribution. At that time, the estimation of liver concentrations was not available. Therefore, when conducting pharmacokinetic studies, it was assumed that hepatocyte concentrations approximate plasma concentrations, the drug equilibration across the sinusoidal membrane being obtained by passive diffusion. With the discovery of hepatocyte transporters that modify the transport rates across the hepatocyte membranes, this assumption is no longer valid. The activity of sinusoidal transporters can be much higher than passive diffusion, increasing the hepatocyte concentrations over the plasma concentrations. Moreover, the drug concentrations generated by the hepatocyte uptake clearances are simultaneously modified by efflux clearances from hepatocytes into bile canaliculi and back into sinusoids. Thus, depending on the relative hepatocyte influx and efflux clearances, drug hepatocyte concentrations can exceed, equal, or be lower than plasma concentrations. Disconnection between the hepatocyte and plasma concentrations is even more unpredictable when the expression and/or function of membrane transporters are altered.

With recent advances in liver imaging, the estimation of liver concentrations is now possible following the injection of hepatobiliary contrast agents and radiotracers. To study more specifically the activity of hepatocyte transporters *in vivo*, radiotracers for positron emission tomography (PET) and single-photon emission computed tomography (SPECT) imaging and gadolinium complexes for magnetic resonance imaging (MRI) can be injected before the image acquisition [1–4]. However, how these images are generated remains partially unknown. Moreover, most experiments that would be helpful to increase this understanding cannot be performed *in vivo*. For these reasons, we investigated the liver distribution of the magnetic resonance (MR) contrast agent gadobenate dimeglumine (Gd-BOPTA, MultiHance®, Bracco Imaging) in isolated perfused rat livers (IPRLs).

In contrast to cultured cells, IPRL maintains the liver architecture and conserves the lobular distribution of drug transporters and metabolising enzymes. All other cells that may interfere with hepatocyte functions are present, and the bile excretion is preserved. Thus, IPRL simulates *in vivo* conditions, while avoiding interferences with other organs. In pharmacology, IPRLs are mainly used to measure pharmacokinetic parameters of injected drug, such as the liver extraction ratios, the synthesis of metabolites by hepatocyte enzymes, and the bile excretion of drugs and their metabolites. Several reviews described the various ways to perfuse rodent livers and the numerous investigations that can be performed with this experimental model [5–7].

In IPRL, we quantify simultaneously the Gd-BOPTA concentrations in each liver compartment and the transfer rates between these compartments. The concentrations were measured either by MR signal intensity or by count rates when the contrast agent was labelled by [^153^Gd]. Although numerous research teams have been using IPRL to measure clearances from sinusoids to bile canaliculi as well as from hepatocytes back to sinusoids, we are the first group that combines these kinetic parameters with the quantification of concentrations. In this review, we show how Gd-BOPTA hepatocyte concentrations are modified by liver temperature and liver flow rates. We define new pharmacokinetic parameters to quantify the multiple resistance-associated protein 2 (Mrp2) function, and present how transfer rates generate the Gd-BOPTA concentrations in rat liver compartments. These findings better explain how liver imaging with hepatobiliary radiotracers and contrast agents is generated and improve the image interpretation by clinicians.

## 2. Methods

### 2.1. How We Perfuse Rat Livers?

Following the anaesthesia of male Sprague Dawley rats, we cannulate the portal vein, the hepatic artery being too small to be perfused. A Krebs–Henseleit bicarbonate (KHB) solution is pumped without delay through the catheter, while the solution is discarded by a vena cava transection following its liver distribution. Liver flow rate is slowly increased over one min up to 30 ml/min. In a second step, the chest is opened and a second cannula is inserted into the right atrium to collect solutions flowing from the hepatic veins. Finally, the abdominal inferior vena cava is ligated allowing all solutions perfused by the portal vein to be eliminated by the hepatic veins. The entire perfusion system includes a reservoir, a pump, a heating circulator, a bubble trap, a filter, and an oxygenator. Solutions of perfusion are equilibrated with a mixture of 95% O_2_-5% CO_2_, allowing normal liver functions during 120 min. Livers are perfused with the KHB buffer ± contrast agents or radiotracers using a non-recirculating system. Thus, livers are perfused with newly prepared solutions throughout the protocol. In each experiment, the common bile duct is cannulated with a thin catheter to collect bile samples and measure the bile flow rates. Perfusate samples are also collected from the hepatic veins.

### 2.2. Properties of Gd-DTPA and Gd-BOPTA

**Figure 1:**
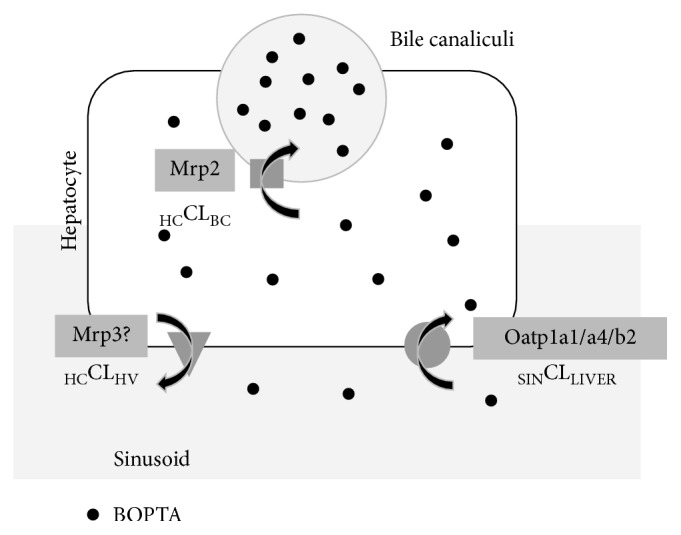
Gd-BOPTA transport across hepatocyte membranes. The MRI contrast agent distributes into sinusoids and interstitium before entry into rat hepatocytes across the organic anion transporting polypeptides (Oatp1a1/a4/b2). Once inside hepatocytes, Gd-BOPTA exits into bile canaliculi (BC) across the multiple resistance-associated protein 2 (Mrp2) or back into sinusoids across the sinusoidal membrane. Gd-BOPTA liver concentrations are estimated by liver MRI or quantified with a gamma counter when the molecule is labelled with ^153^Gd. Sinusoidal clearance to liver (_SIN_CL_LIVER_, ml/min) is [(*C*_PV_–*C*_HV_) × 30]/*C*_PV_, where *C*_PV_ is the concentration (*µ*M) in portal vein and 30 ml/min is the constant liver flow rates. The clearance from hepatocytes to bile canaliculi (_HC_CL_BC_, ml/min) is (*C*_BC_ × bile flow rates)/*C*_HC_, where *C*_BC_ is the Gd-BOPTA concentration in bile canaliculi and *C*_HC_ is the Gd-BOPTA concentration in hepatocytes. The clearance from hepatocytes to interstitium (_HC_CL_INT_, ml/min) is (*C*_HV_ × 30)/*C*_HC_, where *C*_HV_ is the Gd-BOPTA concentration in the hepatic veins. The Gd-BOPTA concentrations (illustrated by the number of circles) increase from sinusoids, to hepatocytes, and to bile canaliculi.

Gadopentetate dimeglumine (Magnevist®, Bayer Pharma, Gd-DTPA) is a MRI contrast agent that diffuses exclusively into the extracellular space of the liver [8]. The hepatocyte uptake and bile excretion are negligible, and its overall body excretion occurs by glomerular filtration. In contrast, following an intravenous injection, Gd-BOPTA distributes into the extracellular space and enters into hepatocytes [9]. This contrast agent is highly soluble in water and exhibits a weak plasma protein binding (<5%) that is efficient enough to increase the MR signal intensity in plasma and tissues [10]. Gd-BOPTA is not metabolised in hepatocytes. In *Xenopus laevis* oocytes, we show that the rat sinusoidal transporters of Gd-BOPTA are the organic anion transporting polypeptides 1a1 (Oatp1a1), Oatp1a4, and Oatp1b2 [11] (Figure 1). Following the study of de Haën et al. [12], we confirmed that Gd-BOPTA is transported into bile canaliculi through the multiple resistance-associated protein 2 (Mrp2) [11]. In liver perfused from rats lacking Mrp2, no Gd-BOPTA is present in bile samples.

### 2.3. Perfusion of Contrast Agents and Radiotracers

**Figure 2:**
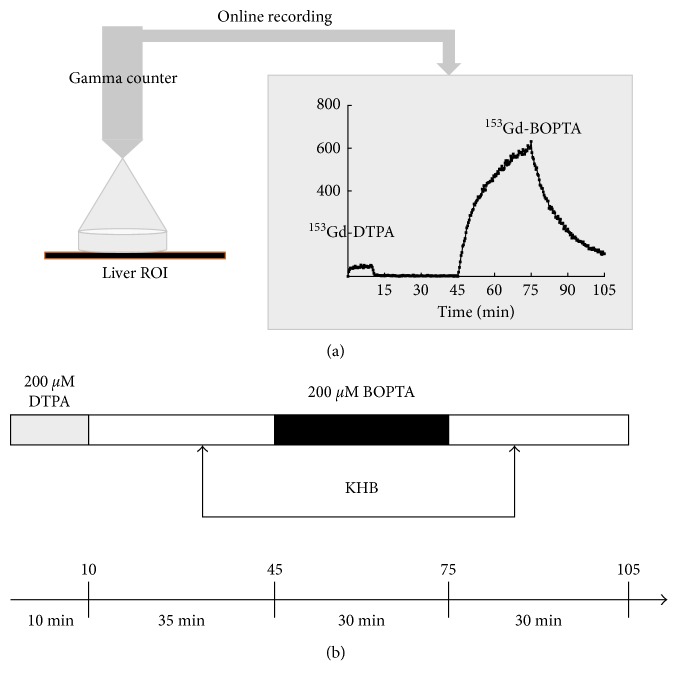
Protocol of the isolated perfused rat livers. (a) A gamma counter detects online the count rates coming from a region of interest (ROI) delineated within a rat liver lobe. The system avoids traumatic collection of small biopsies over time. (b) We standardise the perfusion protocol to compare results in various experimental conditions. Rat livers are perfused successively with Gd-DTPA that distributes into the extracellular space and Gd-BOPTA that enters into hepatocytes after extracellular distribution. Between drug perfusions, Krebs–Henseleit bicarbonate (KHB) solutions are perfused (rinse periods).

Over the years, the perfusion protocol has been standardised to compare the results in various experimental conditions. Gd-DTPA is perfused during 10 min at the beginning of the protocol to quantify the liver concentrations generated by its distribution into the extracellular space (Figure 2). Then, a KHB solution rinses the liver from Gd-DTPA. Gd-BOPTA is perfused during 30 min to quantify its accumulation into the extracellular space and hepatocytes. Livers are finally rinsed with KHB solution during 30 min. Thus, two periods are distinguished: (1) the Gd-BOPTA perfusion period that evidences how the contrast accumulates in the liver compartments, and (2) the Gd-BOPTA rinse period that investigates how the contrast leaves hepatocytes into bile canaliculi and back into sinusoids. To quantify both contrast agents with the gamma counter, we add ^153^GdCl_3_ to 0.5 *µ*M Gd-BOPTA or Gd-DTPA solutions (1 MBq/ml). ^153^Gd-DTPA and ^153^Gd-BOPTA are diluted in KHB solution to obtain 200 *μ*M solutions. Unlabelled Gd-DTPA and Gd-BOPTA are used when imaging is performed in the MRI room. In these last experiments, 500 *µ*M Gd-DTPA and Gd-BOPTA are perfused.

### 2.4. Quantification of Gd-BOPTA Concentrations in Livers and Hepatocytes

In livers perfused with unlabelled Gd-DTPA and Gd-BOPTA, the only available parameter is the signal intensity enhancement, similarly to clinical studies. Transverse images are acquired in a 1.5 T MR system with a fast gradient-echo T1-weighted MR sequence. A region of interest is drawn on the short-axis view of the liver, excluding all large vessels. The region of interest remains constant during the entire experiment. However, the MRI room is not a user-friendly environment and sample collection is limited. To overcome this disadvantage, we set up the IPRL in a laboratory dedicated to experiments with radioactivity. To quantify the liver concentrations of ^153^Gd-DTPA and ^153^Gd-BOPTA, a gamma counter that measures radioactivity online is placed 1 cm above rat liver (Figure 2). The counter measures the radioactivity in a region of interest inside the same liver lobe in each experiment. To transform the radioactivity count rates measured by the counter into ^153^Gd-BOPTA concentrations, the radioactivity contained in the liver at the end of each experiment is measured and related to the last count rates detected by the counter. The concentrations in the common bile duct and the hepatic veins are measured every 5 min in a gamma counter. Concentrations are expressed in *µ*M in the great vessels, the bile duct, and the livers.

The gamma counter placed over the liver lobe quantifies simultaneously count rates originating from sinusoids, interstitium, bile canaliculi, and hepatocytes. To estimate the ^153^Gd-BOPTA concentrations in bile canaliculi, we multiply the bile concentrations measured in the common bile duct by 0.43%, which corresponds to the relative volume of bile canaliculi according to Blouin et al. [13]. Knowing Gd-BOPTA concentrations in bile canaliculi and extracellular compartment (estimated by Gd-DTPA concentrations), we subtract both values from the Gd-BOPTA total liver concentrations to obtain Gd-BOPTA concentrations in the volume of hepatocytes within the region of interest. Finally, because Gd-BOPTA concentrations originate from a 78% volume of hepatocytes, we must increase the values to 100% to obtain the true hepatocyte concentrations [13].

### 2.5. Transfer Rates across Hepatocyte Membranes

In IPRL, we can easily measure the transfer rates across hepatocyte transporters (Figure 1) [14]. Elimination from sinusoids to livers (_SIN_ELIMR_LIVER_, nmol/min) is measured by (*C*_PV_–*C*_HV_) × 30 (constant portal blood flow, ml/min), and sinusoidal clearance to liver (_SIN_CL_LIVER_, ml/min) is _SIN_ELIMR_LIVER_/*C*_PV_, where *C*_PV_ is the Gd-BOPTA concentration perfused via the portal vein and *C*_HV_ is the Gd-BOPTA concentrations measured in the hepatic veins. The transfer rates from hepatocytes to bile canaliculi or interstitium are also measured. _HC_ELIMR_BC_ (nmol/min) is *C*_BC_ × bile flow rate, where *C*_BC_ is the Gd-BOPTA concentration in bile canaliculi. The _HC_ELIMR_INT_ (nmol/min) is *C*_HV_ × liver flow rate, where *C*_HV_ is the concentrations in hepatic veins. The _HC_CL_BC_ (_HC_ELIMR_BC_/*C*_HC_) and _HC_CL_HV_ (_HC_ELIMR_HV_/*C*_HC_) are also calculated, where *C*_HC_ is the Gd-BOPTA concentration in hepatocytes.

### 2.6. Canalicular Transport Activity of Mrp2

To assess Mrp2 function, we calculate the Gd-BOPTA gradients between the bile and hepatocyte concentrations over time (Figure 5(c)). Another way to illustrate Gd-BOPTA canalicular transport through Mrp2 is to plot its hepatocyte concentrations (*x*-axis) and its bile concentrations (*y*-axis) (Figure 5(d)) [15]. To analyse these nonlinear regression curves, we fit the experimental values with the equation *A* + *BX* + *CX*^2^, where *A* is the intercept on the *y*-axis, *B* is the slope of regression, and *C* is the plateau at maximal concentrations. Finally, to understand whether the canalicular fluid transport, the Gd-BOPTA bile concentrations, or both interfere with the Gd-BOPTA excretion rates into bile, we plot the bile excretion rates (*x*-axis) with the bile flow rates (*y*-axis), as previously published (Figure 5(b)) [16]. For these investigations, we perfused rat livers with the single-photon emission computed tomography radiotracers ^99m^Tc-DTPA (TechneScan DTPA®, b.e.imaging, Schwyz, Switzerland) and Mebrofenin (^99m^Tc-MEB, Choletec®, Bracco Imaging) [17–19]. MEB is transported from sinusoids into bile canaliculi by the same transporters as Gd-BOPTA, but its extraction ratio is much higher (94%) than that of Gd-BOPTA (8%). DTPA is labelled with ^99m^Tc (25 mg, 7 MBq), while MEB is labelled with ^99m^Tc (40 mg, 11 MBq). DTPA and MEB are diluted to obtain 64 *μ*M concentrations. Thus, we can compare the Mrp2 function of one contrast agent and one radiotracer that display the same hepatocyte transport but different pharmacokinetics.

## 3. Experimental Results

### 3.1. Gd-BOPTA Concentrations in Normal Livers

**Figure 3:**
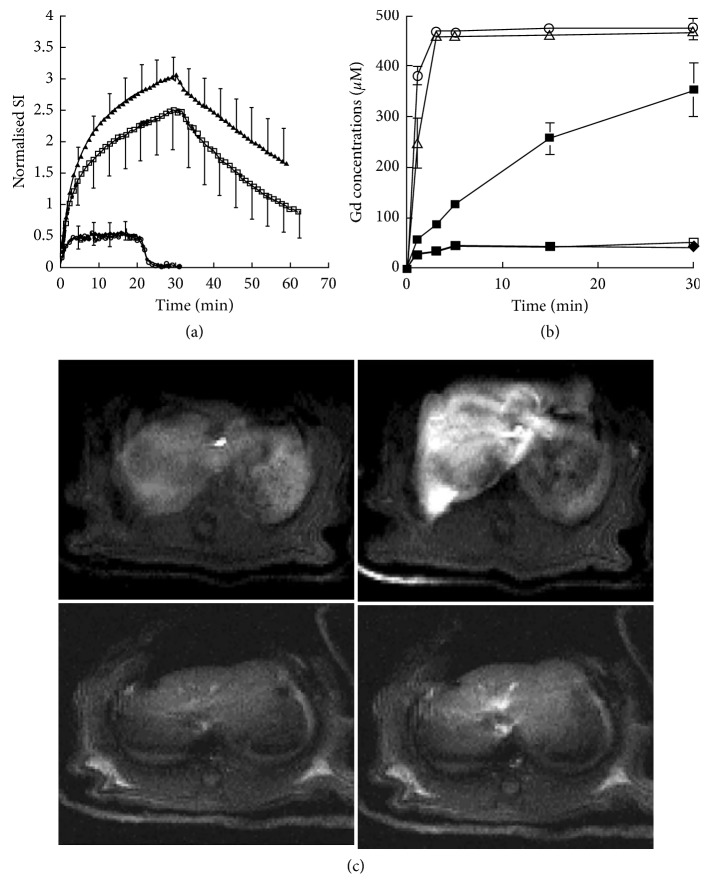
Gd-BOPTA distribution in rat livers. (a) The livers are perfused with 500 *µ*M Gd-DTPA (♦, *n* = 4 ), 500 *µ*M Gd-BOPTA (□, *n* = 4), 500 *µ*M Gd-BOPTA and 500 *µ*M bromosulfophthalein (BSP, ◯, *n* = 4). An additional group of rats with acute bile duct ligation is perfused with 500 *µ*M Gd-BOPTA (▲, *n* = 2). BSP perfusion prevented Gd-BOPTA uptake. (b) Gadolinium concentrations in portal veins (◯), hepatic veins (△), and hepatic tissues (■) during Gd-BOPTA perfusion. Additional livers are perfused with Gd-DTPA (●) and Gd-BOPTA + BSP (□). Three liver biopsies are collected at each time point. (c) Representative MR images of a liver perfused with Gd-DTPA and Gd-BOPTA (upper panels) or DTPA and Gd-BOPTA + BSP (lower panels). Adapted from [20] with permission.

In the initial study, we measure the liver signal intensity during the perfusion of Gd-DTPA and Gd-BOPTA (Figure 3) [20]. Liver enhancement with Gd-DTPA rapidly reaches a steady state, while liver enhancement increases over the 30 min perfusion period (Figure 3). During the rinse period, the signal intensity does not return to baseline values as observed with Gd-DTPA. Pharmacokinetic modelling estimates that the half-lives of Gd-DTPA entry and exit are identical (1.3 ± 0.9 min) and shorter than those observed with Gd-BOPTA (4.8 ± 0.3 min for entry and 17.5 ± 2.8 min for exit). Interestingly, the coperfusion of the dye bromosulfophthalein (BSP) with Gd-BOPTA prevents the contrast agent entry into hepatocytes and the mean half-lives are similar to those measured with Gd-DTPA (Figures 3–3). This BSP inhibition of hepatocyte Gd-BOPTA uptake is the first drug-drug interaction evidenced by liver imaging. Acute bile duct ligation at the time of surgery does not interfere with Gd-BOPTA uptake but slows down the decreased signal intensity during the rinse period (Figure 3). These MR results are confirmed when we measure Gd concentrations by inductively coupled plasma atomic emission spectrometry in liver biopsies (Figure 3). In the portal and hepatic veins, the concentrations remain close to 500 *µ*M at steady state.

These initial experiments demonstrate that contrast agents can be successively imaged in IPRL. The Gd-DTPA perfusion estimates the signal intensity increase associated with the extracellular volume distribution. According to its fast and steady distribution, additional signal intensity observed during Gd-BOPTA perfusion relates to the hepatocyte entry and the transfer into bile canaliculi. Because contrast agents are perfused with a KHB containing no protein, protein binding does not alter the Gd-BOPTA entry. None of the contrast agents modifies the portal pressure or the hepatic O_2_ consumption [20].

### 3.2. Gd-BOPTA Concentrations in Livers with Chronic Biliary Cirrhosis

We then investigate the signal intensity enhancement during Gd-BOPTA perfusion in livers isolated from rats that had a bile duct ligation (BDL) 15, 30, and 60 days before the liver isolation [21]. BDL induces a severe hepatic injury that increases over time with a downregulation of the Gd-BOPTA transporter expression. The extracellular space (assessed by Gd-DTPA imaging) significantly increases with the disease severity. The Gd-BOPTA-induced signal intensity enhancements are similar in control rats, BDL-15 rats, and BDL-30 rats, but decrease significantly in severe cirrhosis (BDL-60 rats). Thus, Gd-BOPTA-induced signal intensity enhancements cannot be related to the transporter expression, emphasising the need for the simultaneous quantification of hepatocyte concentrations and transfer rates across membranes.

### 3.3. Role of Liver Temperature on Gd-BOPTA Liver Concentrations

**Figure 4:**
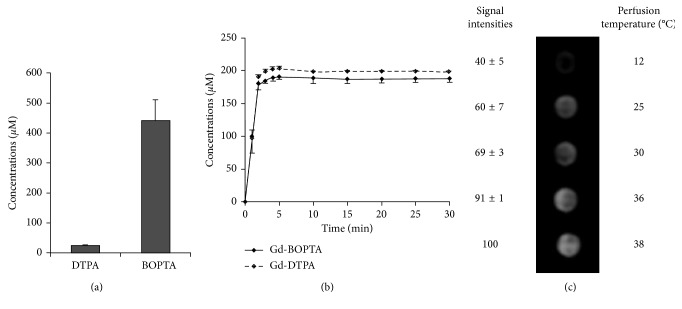
Role of liver temperature on Gd-BOPTA liver concentrations. (a) ^153^Gd-DTPA and ^153^Gd-BOPTA concentrations after a 30 min perfusion (200 *µ*M, 38°C). (b) Time course of contrast agent concentrations in hepatic veins (mean ± SD, *n* = 3). (c) MR images of tubes containing biopsies of livers perfused during 30 min with Gd-BOPTA (200 *µ*M) at 12, 25, 30, 36, and 38°C. The signal intensity of livers perfused at 38°C is 100%. Data are mean ± SD (*n* = 3). Adapted from [22] with permission.

In these experiments, normal livers are perfused with ^153^Gd-DTPA (200 *µ*M) and ^153^Gd-BOPTA (200 *µ*M) at 12, 25, 30, 36, and 38°C to collect samples in the hepatic veins over time (Figure 4(b)) [22]. At the end of the perfusion, liver biopsies are imaged by MRI. The liver ^153^Gd-DTPA and ^153^Gd-BOPTA concentrations are also measured with a gamma counter (Figure 4(a)). A low amount of Gd-BOPTA taken up by hepatocytes (3%) explains the high Gd-BOPTA concentrations measured in hepatic veins (Figure 4(b)). Nevertheless, the maximal Gd-BOPTA concentrations (188 ± 5 *µ*M) in hepatic veins are significantly lower than those of Gd-DTPA (200 ± 1 *µ*M). The MR images of liver biopsies show that the Gd-BOPTA accumulation into hepatocytes declines with temperatures (Figure 4(c)). At 12°C, Gd-BOPTA does not enter into hepatocytes and behaves as Gd-DTPA. Temperature variation from 36 to 38°C significantly increases Gd-BOPTA hepatocyte accumulation, suggesting that these body temperatures may interfere with imaging in patients.

In these preliminary studies, we show that IPRL is an interesting model to quantify liver concentrations of imaging radiotracers and contrast agents and to assess their temperature dependence. By substituting ^153^Gd in the Gd-BOPTA molecule, we quantify accurately Gd-BOPTA liver concentrations. However, at that time, we collected liver biopsies for this quantification and few data were available. In the following studies, we place a gamma counter over the liver to record online the accumulation of radiotracers in the livers (Figure 2).

### 3.4. Canalicular Transport of Gd-BOPTA and MEB across Mrp2

Bile excretion of compounds is an important liver function. Primary bile forms at canalicular membrane of adjacent hepatocytes and transports endogenous (bile salts and bilirubin) as well as exogenous (imaging) compounds [23, 24]. Most compounds that entered hepatocytes cross the canalicular membrane by export pumps. This primary bile is then modified along ductules and ducts by absorptive and secretory processes that take place in the cholangiocyte epithelium [23].

In the IPRL, we investigate two new parameters to assess Mrp2 transport function: the gradients between Gd-BOPTA hepatocyte and bile concentrations over the perfusion period, and a unique parameter named canalicular concentration ratio (CCR) that represents the slope of the nonlinear regression curve between hepatocyte and bile concentrations [15].

#### 3.4.1. Changes of Bile Flow Rates with Hepatobiliary Compounds

**Figure 5:**
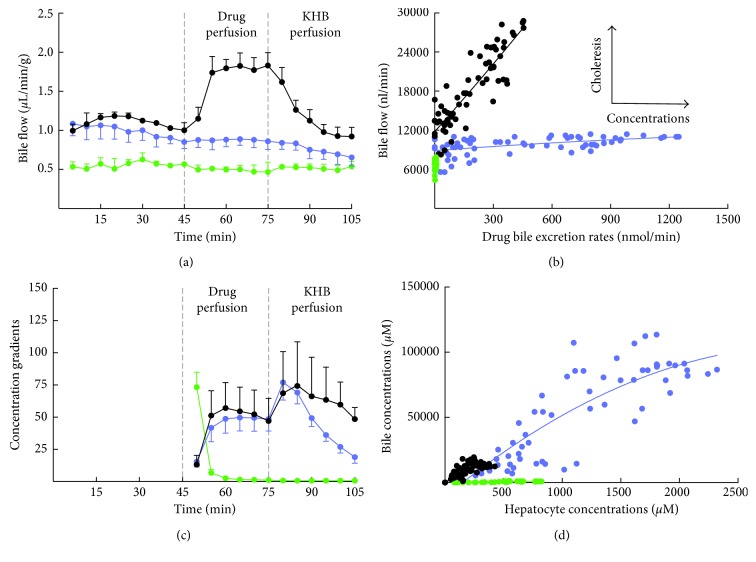
Canalicular transport across Mrp2. (a) Bile flow rates (*µ*l/min/g) during the perfusion of Gd-DTPA, Gd-BOPTA, and MEB. (b) Plots between drug bile excretion rates (*x*-axis, nmol/min) and bile flow rates (*y*-axis, nl/min). (c) Concentration gradients between bile canaliculi and hepatocytes over time. (d) Plots between drug hepatocyte concentrations (*x*-axis, *µ*M) and drug bile concentrations (*y*-axis, *µ*M). Three groups of rat livers are perfused: (1) livers isolated from normal rats and perfused with 200 *μ*M Gd-DTPA and 200 *μ*M Gd-BOPTA (black circles, *n* = 5); (2) livers isolated from rats lacking Mrp2 and perfused with 200 *μ*M Gd-DTPA and 200 *μ*M Gd-BOPTA (green circles, *n* = 3); and (3) livers isolated from normal rats and perfused with 64 *μ*M DTPA and 64 *μ*M MEB (blue circles, *n* = 5). Adapted from [15] with permission.

We show that Gd-DTPA perfusion does not modify the bile flow rates (Figure 5(a)). The contrast agent does not enter into hepatocytes and has no bile excretion. Bile flow rates do not change during MEB perfusion, but importantly increase during Gd-BOPTA perfusion. The choleretic effect of Gd-BOPTA is linked to bile concentrations [25]. The bile flow rates return to basal values during the rinse period, according to the gradual decrease of Gd-BOPTA hepatocyte concentrations. To understand whether the canalicular fluid transfer, the bile concentrations, or both interfere with drug excretion rates into bile, we plot the drug bile excretion rates (*x*-axis) and the bile flow rates (*y*-axis) as previously published [16] (Figure 5(b)). The regressions clearly show that the bile excretion rates of MEB are driven by the bile concentrations, while the Gd-BOPTA bile excretion rates increase according to both bile concentrations and fluid transfer.

#### 3.4.2. Canalicular Transport Activity across Mrp2

The Gd-BOPTA and MEB gradients between hepatocyte and bile concentrations over the perfusion period rapidly increase to reach a similar plateau (Figure 5(c)). However, during the rinse period, the gradients have different evolution. By plotting the hepatocyte concentrations (*x*-axis) and the bile concentrations (*y*-axis), we find similar regression slopes (CCR) for Gd-BOPTA and MEB (Figure 5(d)). Thus, with these parameters, we can assess the Mrp2 function independently from the cellular uptake of compounds. The shape of these nonlinear regressions confirms that the transport via Mrp2 is saturated at high concentrations (Figure 5(d)). CCR is null in rat livers deficient in Mrp2. CCRs might also be a parameter of Mrp2 inhibition, but we did not yet quantify these data.

### 3.5. How Transfer Rates Generate Gd-BOPTA Concentrations in Rat Liver Compartments

More recently, we measured the true concentrations in hepatocytes and show how transfer rates across sinusoidal and canalicular membranes generate these concentrations without any pharmacokinetic modelling (Figure 1) [14]. Liver parenchyma is divided into sinusoids, interstitium, hepatocytes, and bile canaliculi. In clinical MRI, the averaged signal intensity is quantified in regions of interest, but concentrations from each compartment cannot be estimated. In the common bile duct, the signal intensity estimates true concentrations because the structure has a single compartment. In portal and hepatic veins, they estimate true concentrations once cell volumes are subtracted. In human livers, transfer rates between two liver compartments can only be estimated by pharmacokinetic modelling [26–30]. However, it is important to measure true concentrations inside each compartment, because concentrations across membranes partly regulate transfer rates across them [31].

**Figure 6:**
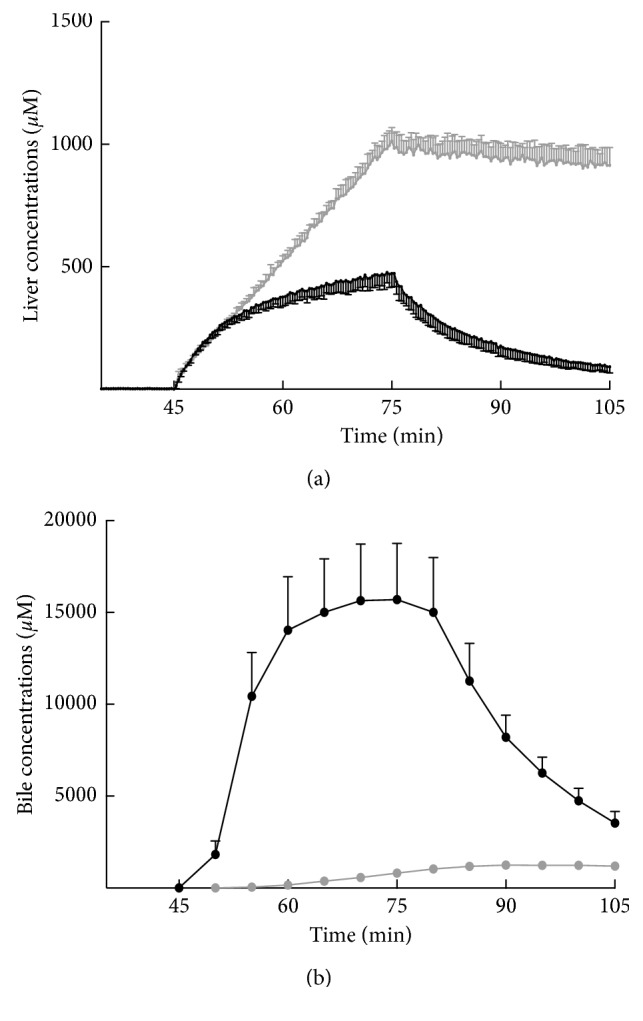
Gd-BOPTA concentrations over time in structures identified at clinical MRI. Values are either liver concentrations assessed by the gamma counter (a) or true concentrations in bile (b). Gd-BOPTA (200 *μ*M) is perfused from 45 to 75 min and replaced by a rinse solution from 75 to 105 min. Two groups of rats are studied: normal rats (*n* = 5, black symbols) and rats lacking Mrp2 (n = 3, grey symbols). Adapted from [14] with permission.

In normal livers, Gd-BOPTA concentrations increase rapidly over 10 min (Figure 6(a)) [14]. Thereafter, the increase is slower and the concentrations reach 473 ± 56 *μ*M. When Gd-BOPTA perfusion is replaced by the KHB solution, the concentrations steadily decrease. The Gd-BOPTA concentrations in common bile duct (Figure 6(b)) are present 5 min after the start of perfusion (first sampling), and the maximal true concentrations reach 15700 ± 3100 *μ*M (end of perfusion), a concentration 78 times higher than that perfused in portal veins (200 *μ*M). In rats lacking Mrp2, liver concentrations linearly increase during the perfusion period (Figure 6(a)). The concentrations are much higher in livers lacking Mrp2 than in normal livers, while Gd-BOPTA concentrations in the common duct are tiny (Figure 6(b)). In this group, Gd-BOPTA remains trapped inside hepatocytes until the end of the protocol.

**Figure 7:**
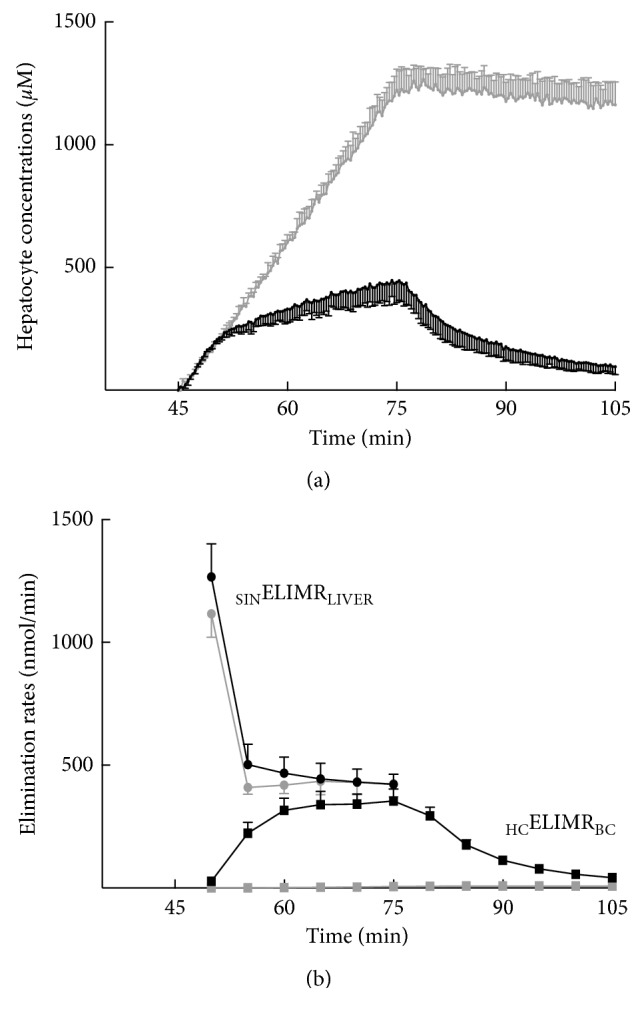
Gd-BOPTA concentrations in hepatocytes over time (a) according to the concomitant Gd-BOPTA elimination rates from sinusoids to liver (_SIN_ELIMR_LIVER_, nmol/min) and Gd-BOPTA elimination rates from hepatocytes to bile canaliculi (_HC_ELIMR_BC_) (b). Two groups of rats are studied: normal rats (*n* = 5, black symbols) and rats lacking Mrp2 (*n* = 3, grey symbols). Livers are perfused with 200 *μ*M Gd-BOPTA (45 to 75 min) and rinse solution (75 to 105 min). Adapted from [14] with permission.

Finally, we can explain how Gd-BOPTA transfer rates generate concentrations in normal hepatocytes (Figures 7(a) and 7(b)). At the beginning of perfusion, the Gd-BOPTA elimination from sinusoids to livers (_SIN_ELIMR_LIVER_) is much higher than the elimination rates from hepatocytes to bile canaliculi (_HC_ELIMR_BILE_). Then, the _SIN_ELIMR_LIVER_ decreases but remains higher than the _HC_ELIMR_BILE_, and the hepatocyte concentrations continue to increase moderately. The absence of _HC_ELIMR_BILE_ clearly explains the high hepatocyte concentrations in livers lacking Mrp2.

### 3.6. Hepatocyte Transport Activity and Liver Perfusion

**Figure 8:**
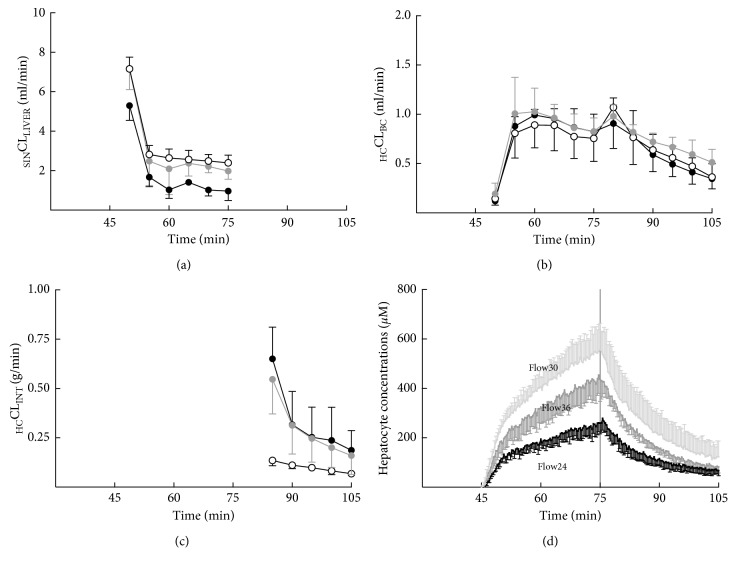
Hepatocyte transport activity and liver perfusion. Gd-BOPTA hepatocyte concentrations (d) over time according to clearances from sinusoids to liver (_SIN_CL_LIVER_, a), clearances from hepatocytes to bile canaliculi (_HC_CL_BC_, b), and clearances from hepatocytes back to interstitium (_HC_CL_INT_, c). Livers are perfused with 200 *µ*M Gd-BOPTA (45 to 75 min) and Krebs–Henseleit bicarbonate (KHB) solution (75 to 105 min) at various flow rates: 24 ml/min (black circles), 30 ml/min (white circles), or 36 ml/min (grey circles). Adapted from [32] with permission.

To investigate whether changing liver flow rates modifies Gd-BOPTA hepatocyte concentrations, we isolate livers from normal rats and perfused them at various liver flow rates: 24 ml/min (*n* = 3, Flow24), 30 ml/min (*n* = 5, Flow30), or 36 ml/min (n = 3, Flow36) [32]. Of note, the standard liver flow rate is 30 ml/min. The perfused concentration in portal veins is 200 *µ*M, and changing liver flow rates modifies Gd-DTPA and Gd-BOPTA delivery rates from 4800 nmol/min (Flow24), 6000 nmol/min (Flow30), and 7200 nmol/min (Flow36). Portal pressures increase with liver flow rates, while bile flow decreases when livers are perfused at the low flow rate. High (Flow36) and low (Flow24) flow rates decrease hepatocyte Gd-BOPTA concentrations in comparison with the standard flow rate (30 ml/min, Figure 8). These low hepatocyte concentrations are explained by the low _SIN_CL_LIVER_ (uptake function) and the high _HC_CL_INT_ (efflux back), _HC_CL_BC_ being identical in the 3 groups. Consequently, changing portal flow rates modifies Gd-BOPTA hepatocyte concentrations, a result important to consider when interpreting liver imaging.

## 4. Conclusion

Our investigations clearly show that MR contrast agents can be imaged in IPRL. With our experimental protocol, the Gd-DTPA perfusion estimates the signal intensity increase associated with the extracellular volume distribution. According to its fast and steady distribution, additional signals observed during Gd-BOPTA perfusion relate to the hepatocyte entry and the transfer into bile canaliculi. In rats with chronic liver diseases, the Gd-BOPTA-induced enhancements are not related to the modified expression of Gd-BOPTA transporters, emphasising the need for the simultaneous quantification of the hepatocyte concentrations and the transfer rates across cellular membranes in IPRL. In an early study, we assessed the temperature dependence of Gd-BOPTA liver concentrations. We also show that Mrp2 transport function is well characterised by new parameters that are independent of Gd-BOPTA uptake into hepatocytes. More recently, we demonstrate how the transfer rates across sinusoidal and canalicular membranes generate hepatocyte concentrations, and that the liver flow rates alter these concentrations by modifying Gd-BOPTA clearances across the sinusoidal membrane.

IPRL is a convenient model because the experimental conditions are well controlled and simplified. The gamma counter placed over the liver detects the concentrations of imaging compounds, avoiding serial liver biopsies [33]. The delineation of a single region of interest is however a disadvantage in comparison with the 3D liver imaging. The imaging compounds we use are free to enter into hepatocytes because perfused solutions do not contain proteins. It would be easy to add albumin to the perfused solutions to assess the role of protein binding on the uptake clearances. Livers are perfused only through the portal vein, avoiding the complexity of a dual input entry. A flow rate of 30 ml/min (or 1 ml/10 g of body weight) is commonly used when livers are perfused with the KHB solution that contains no red blood cells. To investigate the consequences of liver flow rates on Gd-BOPTA hepatocyte concentrations, we decrease the liver flow rate only to 24 ml/min to avoid liver ischemia. Indeed, we previously published that decreasing liver O_2_ delivery is associated with an increased O_2_ extraction ratio to maintain a normal O_2_ consumption until a threshold where O_2_ consumption decreases with altered hepatic functions [34]. At 24 ml/min, the bile flow is already altered. Finally, we can measure concentrations in all liver compartments and no pharmacokinetic modelling is necessary to calculate Gd-BOPTA transfer rates across hepatocyte membranes.

The new understanding summarised in the present article was directly translated to human liver imaging in two recent publications. In 2016, we reviewed all the published studies that correlate the hepatobiliary MR imaging and the expression of hepatocyte transporters in human hepatocellular carcinomas [35]. We showed how the understanding of signal intensity in these focal lesions relies on the transport function of the human Mrp2. More recently, we published the pharmacokinetic modelling of liver images to quantify the liver perfusion and hepatocyte transport function of patients with chronic liver diseases [36].

## Abbreviations

Gd-BOPTA: Gadobenate dimeglumine, MultiHance®, Bracco Imaging
CCR: Canalicular concentration ratio
Gd-DTPA: Gadopentetate dimeglumine, Magnevist®, Bayer Pharma
^99m^Tc-DTPA: TechneScan DTPA®, b.e.imaging, Schwyz, Switzerland
IPRL: Isolated perfused rat liver
MEB: Mebrofenin, Choletec®, Bracco Imaging
MRI: Magnetic resonance imaging
SPECT: Single-photon emission computed tomography.
